# A Scoping Review of Delphi Methodologies in Aging Research: 2018-2024

**DOI:** 10.1093/geroni/igaf122.515

**Published:** 2025-12-31

**Authors:** Shannon Jarrott, Aaron Ogletree, Shelbie Turner, Michelle Demetres

**Affiliations:** The Ohio State University, Columbus, Ohio, United States; Virginia Tech, Blacksburg, Virginia, United States; Weill Cornell Medicine, New York, New York, United States; Weill Cornell Medicine, New York, New York, United States

## Abstract

The Delphi method engages experts to develop, refine, or establish face validity of materials, such as clinical guidelines or assessment tools. It is a convenient, efficient means of gathering input that also mitigates power dynamics that might exist among varied stakeholders. The number of Delphi studies is growing, and researchers in aging apply the methodology across a range of topics. However, Delphi methodology standards and reporting guidelines vary, potentially limiting study rigor and reproducibility. We conducted a scoping review of Delphi studies in aging research to (a) characterize methodological practices adopted by researchers and (b) identify quality indicators for Delphi studies specific to aging research. Searching multiple databases for published literature from January 2018 through November 2024, we identified 89 eligible studies and extracted key information reflecting recommended Delphi method practices (e.g., pre-identifying consensus). Results demonstrate variation across adopted practices and highlight a need for greater alignment with quality indicators. Percentage of studies adopting the recommended practices ranged from a low of 11% (specifying a target number of expert participants) to a high of 92% (providing the final product). For example, Diamond and colleagues recommended that number of rounds be determined a priori, but this occurred in only 28% of examined studies. We propose a Delphi quality indicator, or criteria, rating form for aging research based on these findings. The criteria rating form can be used to plan and implement the Delphi method and evaluate the trustworthiness of studies and resultant products.

